# Effects of Omega-3 and Omega-9 Fatty Acid Supplementation on HOMA-IR and Inflammatory Markers in Children with Obesity and Insulin Resistance: A Triple-Blind RCT with Post-Intervention Follow-Up

**DOI:** 10.3390/nu18162644

**Published:** 2026-08-13

**Authors:** Javier Alonzo Campos-González, Dana Fernanda Vasquez-Solorio, Samuel Flores-Huerta, Jenny Vilchis-Gil, César Navarro-Hernandez, Carlos Juárez-López, Miguel Klünder-Klünder, Nancy Lucero Martínez-Rodríguez

**Affiliations:** 1 Facultad de Medicina, Universidad Autónoma del Estado de México, Toluca 50180, Mexico; javiercamposmed@gmail.com; 2 Facultad de Medicina, Universidad Nacional Autónoma de México, Mexico City 04510, Mexico; vasquezdana1d@gmail.com; 3 Unidad de Investigación Epidemiológica en Endocrinología y Nutrición, Hospital Infantil de México Federico Gómez, Mexico City 06720, Mexicojvilchisgil@gmail.com (J.V.-G.); 4 Centro Universitario de Occidente, Universidad de Guadalajara, Guadalajara 44100, Mexico; 5 Instituto de Servicios Descentralizados de Salud Pública del Estado de Campeche, Campeche 24040, Mexico

**Keywords:** childhood obesity, insulin resistance, HOMA-IR, omega-3 fatty acids, omega-9 fatty acids, interleukin-6, leptin, randomized controlled trial

## Abstract

**Background/Objectives:** Omega-3 and omega-9 fatty acid supplementation has shown metabolic and immunomodulatory benefits, but evidence in pediatric insulin resistance is limited, and the persistence of effects after treatment discontinuation remains uncharacterized. This study evaluated the independent and combined effects of omega-3 and omega-9 supplementation on the metabolic and inflammatory profile of children with obesity and insulin resistance, during and after active treatment. **Methods:** This prespecified secondary analysis of a multicenter, randomized, triple-blind, three-arm parallel trial (NCT05488223) included 97 children (8–17 years) with overweight/obesity and baseline insulin resistance (HOMA-IR > 3.0). Participants received 1.8 g/day of omega-3 (n = 32), omega-9 (n = 34), or combined supplementation (n = 31) for 3 months, followed by a 2-month post-intervention follow-up. Metabolic and inflammatory markers were assessed at baseline (T1), end of treatment (T2), and follow-up (T3). The primary outcome was the change in HOMA-IR from T1 to T2. **Results:** Active supplementation significantly reduced fasting insulin and HOMA-IR in the Combined (*p* < 0.001) and Omega-9 (*p* = 0.010 and *p* = 0.006) groups; at month 5, only the Combined group maintained significant reductions in both. IL-6 and leptin decreased across all groups during intervention and remained reduced at follow-up. Multivariable regression (n = 96) showed the metabolic response was most strongly associated with baseline metabolic status and concurrent BMI reduction rather than intervention group, which was not an independent predictor of metabolic response, nor consistently of inflammatory outcomes (one exception: a smaller IL-4 increase with omega-9, *p* = 0.043, unconfirmed in GEE). **Conclusions:** Within-group reductions in HOMA-IR and fasting insulin persisted after discontinuation only in the Combined group, but without significant between-group differences at any timepoint, consistent with response magnitude reflecting baseline status and adiposity reduction rather than treatment group per se. This positions combined supplementation as a potential metabolic adjuvant—not a standalone therapy—for HOMA-IR-defined insulin resistance in pediatric obesity, assessed here via anthropometric and biochemical rather than imaging-based measures.

## 1. Introduction

Childhood obesity is a principal driver of early-onset insulin resistance (IR) and cardiometabolic comorbidity. White adipose tissue functions as an active endocrine organ [1,2,3], and in the context of obesity, adipocyte hypertrophy promotes lipoinflammation, characterized by dysregulated adipokine secretion and increased production of proinflammatory cytokines [4,5,6]. This chronic, low-grade inflammatory state represents a central pathophysiological mechanism linking visceral adiposity to impaired insulin signaling in pediatric populations [7].

Omega-3 polyunsaturated fatty acids (PUFAs), particularly eicosapentaenoic acid (EPA) and docosahexaenoic acid (DHA), modulate immune pathways, improve the lipid profile, and have been shown to reduce triglycerides, heart rate, and HOMA-IR in children [8,9,10,11]. Several meta-analyses report improvements in lipid profile and heart rate variability following PUFA supplementation [12,13]; however, longitudinal studies have produced inconsistent results regarding circulating inflammatory mediators and anthropometric outcomes [14]. These discrepancies suggest that clinical response is influenced by dose, intervention duration, and baseline metabolic phenotype.

Omega-9 monounsaturated fatty acids (MUFAs), such as oleic acid, act as metabolic modulators by improving insulin sensitivity and reducing oxidative stress [15,16]. However, evidence on the anti-inflammatory effects of omega-9 remains scarce. A meta-analysis of 31 randomized controlled trials in adults found that oleic acid supplementation significantly reduced C-reactive protein (−0.11; 95% CI: −0.21 to −0.01; *p* = 0.038) but had no effect on other inflammatory markers [17,18].

Mechanistically, omega-3 and omega-9 fatty acids act through distinct but potentially complementary pathways: omega-3-derived EPA and DHA activate GPR120/FFAR4 signaling and modulate proximal insulin signaling and lipotoxicity [9,19], whereas omega-9 (oleic acid) has been shown to improve insulin sensitivity and reduce oxidative stress through complementary metabolic pathways in experimental models [20]. Because these mechanisms converge on overlapping downstream targets—including inflammasome-related regulation of IL-1β secretion and broader metabolic inflammatory signaling [21]—combined supplementation could theoretically produce additive or synergistic effects not captured by studies evaluating either fatty acid in isolation. However, to our knowledge, no prior pediatric trial has directly compared monotherapy against combined omega-3/omega-9 supplementation with post-intervention follow-up, which was the central rationale for this trial’s three-arm design.

Although both fatty acid classes have demonstrated metabolic benefits, existing pediatric trials of omega-3 and/or omega-9 supplementation have primarily reported acute (on-treatment) changes in a limited panel of inflammatory markers (typically CRP, IL-6, and TNF-α), with post-cessation follow-up rarely exceeding the active treatment period itself; broader cytokine panels—including IL-4, IL-1β, IFN-γ, and adipokines such as leptin—combined with a structured post-intervention washout, have not, to our knowledge, been jointly evaluated in a pediatric insulin-resistant population. This combination of a broad inflammatory panel and post-cessation follow-up constitutes the specific evidentiary gap addressed by the present trial. This study therefore evaluated the independent and combined effects of omega-3 and omega-9 supplementation on the metabolic and inflammatory profile of pediatric patients with obesity and insulin resistance, across both active treatment and post-cessation follow-up.

## 2. Materials and Methods

### 2.1. Study Design and Participants

**Figure 1:**
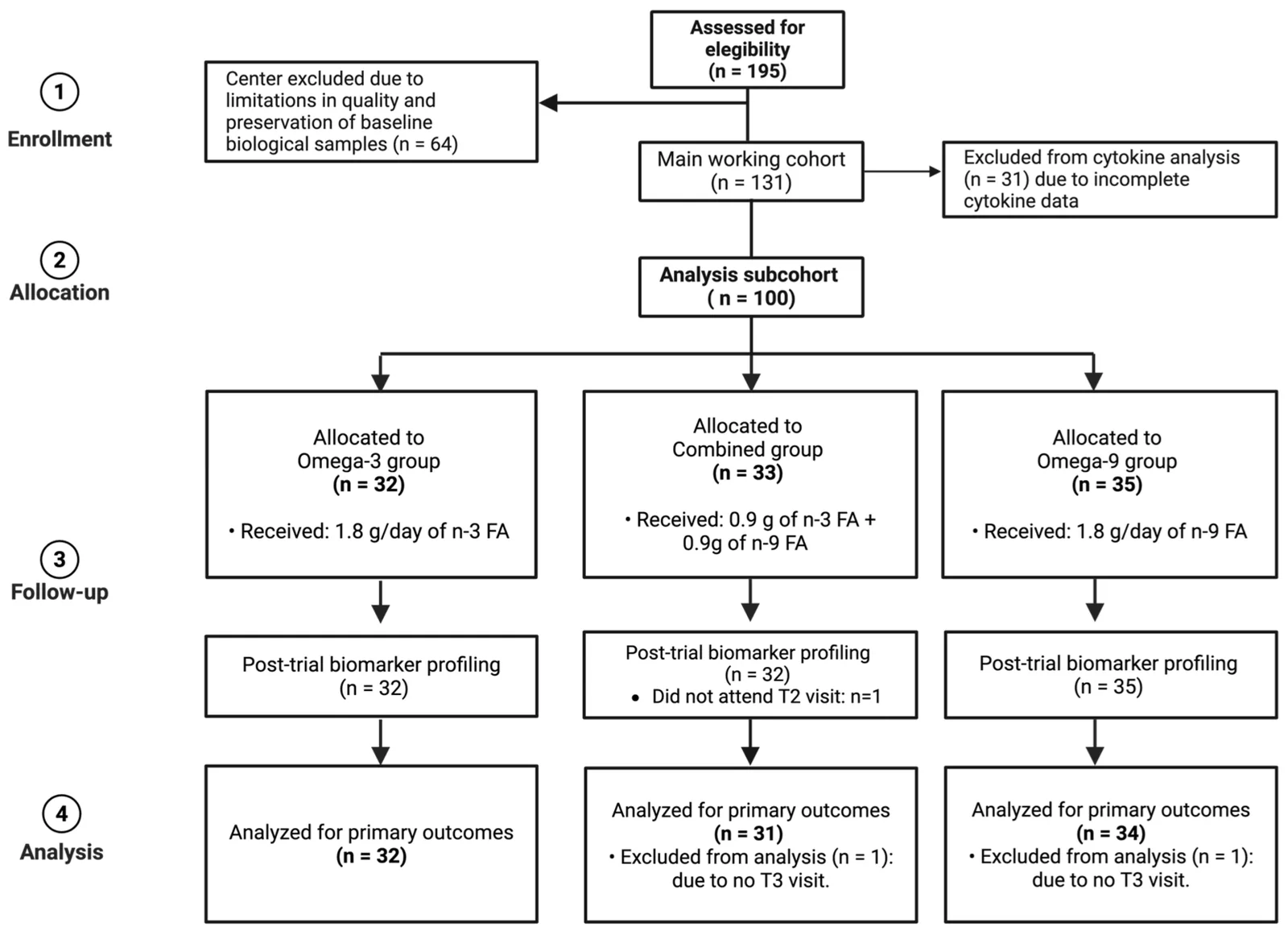
CONSORT flow diagram. Participants from the Guadalajara site (n = 64) were excluded due to limitations in the quality and preservation of baseline biological samples. Of the remaining 131 randomized participants, 31 were excluded from the cytokine analysis sub-cohort due to incomplete cytokine data resulting from technical failures in the processing of stored serum samples. The final analytical sample comprised 97 participants with complete data at all three time points. One Omega-3 participant was excluded from regression models only due to an extreme ΔHOMA-IR value (regression n = 96). T1: baseline; T2: end of treatment (month 3); T3: post-intervention follow-up (month 5). FA: fatty acid; HOMA-IR: homeostatic model assessment of insulin resistance.

This study is a prespecified secondary analysis of a multicenter, randomized, triple-blind, controlled, three-arm parallel clinical trial (NCT05488223). The original protocol comprised a 5-month duration, divided into baseline assessment (T1), 3 months of active intervention (T2), and a 2-month post-intervention follow-up (T3). The initial design included three recruitment sites: Mexico City (Hospital Infantil de México Federico Gómez, HIMFG), the state of Campeche, and Guadalajara (school-based settings). The Guadalajara site (n = 64) was formally excluded from the present analysis due to limitations in the quality and preservation of baseline biological samples required for inflammatory biomarker determination. Following this exclusion, a main working cohort of 131 randomized participants was established, distributed across three intervention arms (omega-3, omega-9, and combined supplementation); randomization methodology followed standard RCT design principles [22]. This secondary analysis derives directly from the original registered protocol (NCT05488223); no separate primary-outcomes publication reporting this trial’s own results has been published to date, and citations to prior omega-3/omega-9 pediatric literature elsewhere in this manuscript (e.g., reference [14]) refer to distinct, earlier studies by overlapping author groups rather than to a prior report of the present trial. After trial completion, a deferred analysis of the inflammatory cytokine profile was performed on serum samples previously aliquoted and stored at −80 °C from each assessment timepoint. This secondary analysis was reported in accordance with the CONSORT 2010 statement, adapted for secondary analyses of randomized trials. The follow-up participants through each stage of the study are presented in Figure 1.

The study population consisted of children aged 8–17 years with a diagnosis of overweight or obesity. Nutritional status was defined using body mass index (BMI)-for-age z-scores (Z-BMI) according to World Health Organization (WHO) criteria, with obesity classified as Z-BMI > +2 standard deviations (SD) [23]. Eligibility for the present analysis required baseline evidence of IR, defined as a Homeostatic Model Assessment of Insulin Resistance (HOMA-IR) > 3.0 [24,25] at T1.

Exclusion criteria included a history of chronic inflammatory, autoimmune, renal, or hepatic disease (except for metabolic dysfunction-associated steatotic liver disease [MASLD]); genetic syndromes; untreated endocrine disorders; and acute infections. Patients receiving pharmacological treatment affecting glucose homeostasis, lipid profile, or inflammatory pathways were also ineligible, as were those who had consumed omega fatty acid supplements or undergone active weight-loss interventions within the 3 months preceding recruitment.

As this is a secondary analysis, analyses were restricted to participants with complete data at all three assessment timepoints (N = 97; omega-3 n = 32, combined n = 31, omega-9 n = 34). Baseline characteristics of the analytical cohort were compared with those of the full randomized cohort (n = 131); no significant differences were identified, making substantial selection bias from this analytical approach unlikely.

### 2.2. Ethical Considerations

This study was conducted under two approved protocols of the Research, Ethics, and Biosafety Committees of the Hospital Infantil de México Federico Gómez: the original trial protocol (HIM/2013/001), under which participants were recruited and randomized, and a subsequent protocol (HIM/2017/109) authorizing the deferred analysis of the inflammatory cytokine profile from stored serum samples. All procedures were conducted in accordance with the ethical principles of the Declaration of Helsinki [26]. Written informed consent was obtained from parents or legal guardians, and informed assent was obtained from pediatric participants prior to enrollment. The trial is registered at ClinicalTrials.gov (NCT05488223). This trial was registered retrospectively (registered on 1 August 2022) after enrollment of the first participant (first participant enrolled on 17 February 2014).

### 2.3. Supplementation Protocol and Adherence

Treatments were isovolumetric and isoenergetic. Participants consumed 1.8 g/day of the assigned lipid intervention for 3 months, divided into two daily doses. To facilitate administration and maintain blinding, each participant received two identically appearing bottles, labeled exclusively as “breakfast” and “dinner.”

Interventions were distributed using combinations of Formula A (marine omega-3 PUFAs; Triple Strength Fish Oil^®^, General Nutrition Corporation [GNC], Pittsburgh, PA, USA; each 0.9 g capsule containing 540 mg eicosapentaenoic acid [EPA; 20:5 *n*-3] and 360 mg docosahexaenoic acid [DHA; 22:6 *n*-3]) and Formula B (avocado oil; Progela, S.A. de C.V., Mexico City, Mexico; each capsule 0.9 g, commercially sourced; independent chromatographic verification of its fatty acid composition and purity beyond manufacturer specification was not performed for this trial), assigned as follows:

- Group 1 (Omega-3 monotherapy): Formula A in both the “breakfast” and “dinner” bottles (total: 1.8 g/day marine PUFAs).
- Group 2 (Combined treatment): Formula A in the “breakfast” bottle and Formula B in the “dinner” bottle (total: 0.9 g/day omega-3 and 0.9 g/day omega-9).
- Group 3 (Omega-9 monotherapy): Formula B in both the “breakfast” and “dinner” bottles (total: 1.8 g/day avocado oil).

All groups received standard age-appropriate nutritional counseling. No caloric restriction or structured exercise programs were implemented. Treatment adherence was monitored by capsule count, with remaining capsules quantified and recorded at each follow-up visit. Adherence was calculated as the percentage of capsules consumed relative to the total target of 180 capsules over the 3-month active phase (2 capsules/day × 90 days).

Tolerability was monitored using a weekly diary card, completed by participants or caregivers, on which daily capsule intake and any symptoms or reactions experienced that day were recorded using a predefined symptom code. No participant discontinued treatment due to tolerability concerns.

### 2.4. Anthropometric and Biochemical Measurements

Anthropometric data were obtained by trained personnel, with participants barefoot and in light clothing. Body weight was measured to the nearest 0.1 kg using a Seca 884 digital scale (Hamburg, Germany), and height to the nearest 0.1 cm using a Seca 225 stadiometer (Hamburg, Germany). Waist circumference (WC) was measured at the midpoint between the lower costal margin and the iliac crest at the end of normal expiration, using a Seca 200 inelastic tape (Hamburg, Germany). Waist circumference percentile was calculated according to age- and sex-specific reference values for Mexican children and adolescents [27].

Venous blood samples were obtained after an 8–12-h fast. Glucose was quantified using the hexokinase method (Dimension RXL Max, Siemens Healthcare Diagnostics, Erlangen, Germany), and insulin was measured by chemiluminescent immunoassay (IMMULITE 1000, Siemens, Erlangen, Germany). Serum concentrations of adiponectin, leptin, interferon gamma (IFN-γ), interleukin-1 beta (IL-1β), interleukin-4 (IL-4), interleukin-6 (IL-6), interleukin-10 (IL-10), monocyte chemoattractant protein-1 (MCP-1), and tumor necrosis factor alpha (TNF-α) were analyzed in duplicate using Merck Millipore multiplex panels (HCYTOMAG-7, HADK1MAG-1, and HADK2MAG-1; MilliporeSigma, Burlington, MA, USA). IR was calculated using the homeostatic model assessment (HOMA-IR = fasting insulin [µIU/mL] × fasting glucose [mg/dL]/405) [28].

Adiponectin was excluded from statistical analyses because 74% of baseline samples fell below the lower limit of detection. Values below or above the detection limits were assigned to the corresponding lower or upper limit. A relevant proportion of baseline leptin concentrations reached the upper detection limit of the assay (600 ng/mL): 19% of samples overall (28% in the omega-3 group, 10% in the combined group, and 18% in the omega-9 group). Leptin was log-transformed in all regression and GEE models, and extreme values are indicated in Table 1. Because these represent instrument-censored values rather than biologically implausible outliers, censored leptin values were retained (assigned to the limit of detection [LOD]) rather than excluded, consistent with standard practice for handling detection-limit-censored biomarkers. As a sensitivity analysis, all leptin models were re-run after excluding participants with values at or above the detection limit (n = 18); results are in Section 3.5.

### 2.5. Statistical Analysis

Statistical analyses were performed on participants with complete data at all three assessment timepoints (N = 97; omega-3 n = 32, combined n = 31, omega-9 n = 34). Continuous variables are reported as mean ± SD or median (interquartile range, IQR), based on Shapiro–Wilk normality testing. Categorical variables are presented as frequencies and percentages.

Net changes (Δ) were calculated as the post-intervention value minus the baseline value. Between-group differences at baseline and in Δ values were assessed using one-way ANOVA or the Kruskal–Wallis test, followed by Bonferroni or Dunn post hoc tests as appropriate. Within-group longitudinal changes were assessed using paired *t*-tests or Wilcoxon signed-rank tests. For longitudinal comparisons of inflammatory biomarkers, the Benjamini–Hochberg false discovery rate (FDR) correction was applied within each variable and period; adjusted *p*-values are reported in Table 2.

The prespecified primary outcome was the change in HOMA-IR from baseline to the third month of intervention (ΔT2 − T1); inflammatory biomarkers constituted secondary outcomes.

Multivariable linear regression models evaluated independent determinants of the metabolic response (Δglucose, Δinsulin, and ΔHOMA-IR) over the short term (ΔT2 − T1) and long term (ΔT3 − T1), using the Combined group as the reference category. Models were performed on n = 96 participants; one Omega-3 participant was excluded from regression models due to an extreme and influential ΔHOMA-IR value (T2 − T1 = +8.45; z = 3.10 relative to the Omega-3 group distribution; Cook’s distance = 1.42, versus a next-highest value of 0.09 among all 97 observations and a conventional threshold of 4/n = 0.04), the only such observation in the dataset. Each model was adjusted for intervention group, age, sex, baseline Z-BMI, concurrent change in Z-BMI, the corresponding baseline outcome value, and total capsule consumption. The ΔZ-BMI coefficient reflects the effect per full unit change in Z-score; given the limited variance of this variable (changes on the order of 0.1 units), its magnitude is large by construction. Given the baseline imbalance observed in TNF-α, the baseline value was included as a covariate in the corresponding analyses to mitigate the risk of regression-to-the-mean bias. Multicollinearity was ruled out using the variance inflation factor (VIF < 2.0). Inflammatory biomarker outcomes were log-transformed prior to regression to meet normality assumptions; model coefficients therefore represent proportional changes (Δlog).

As additional sensitivity analyses, recruitment site (Mexico City/HIMFG vs. Campeche) and pubertal stage (Tanner, prepubertal vs. advanced pubertal, assessed at baseline) were each tested as an additional covariate in the primary short-term HOMA-IR regression model. Recruitment site was balanced across intervention arms (χ^2^ = 0.686, *p* = 0.710), and baseline HOMA-IR did not differ significantly by site (*p* = 0.426), supporting adequate randomization across sites.

**Figure 2:**
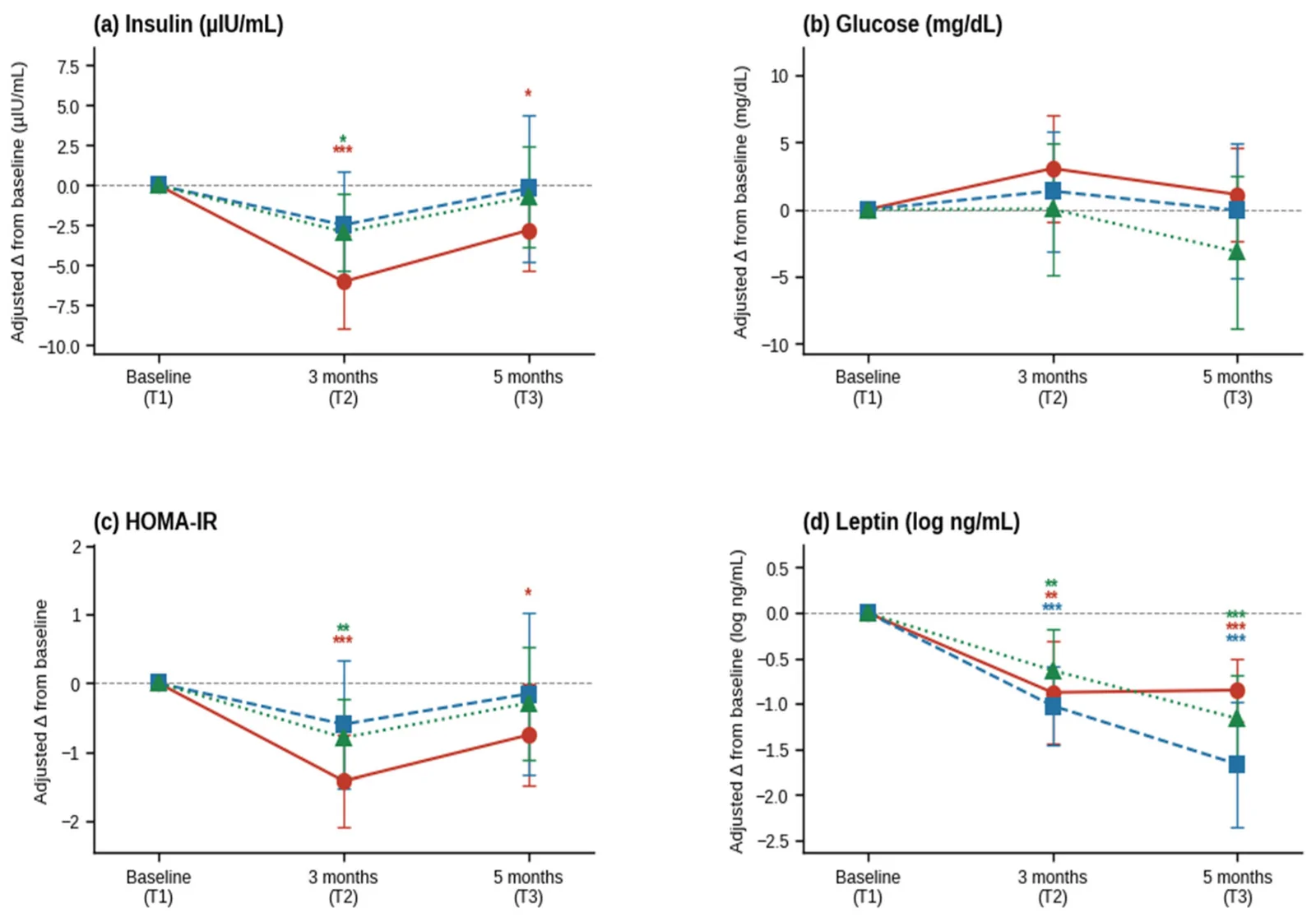

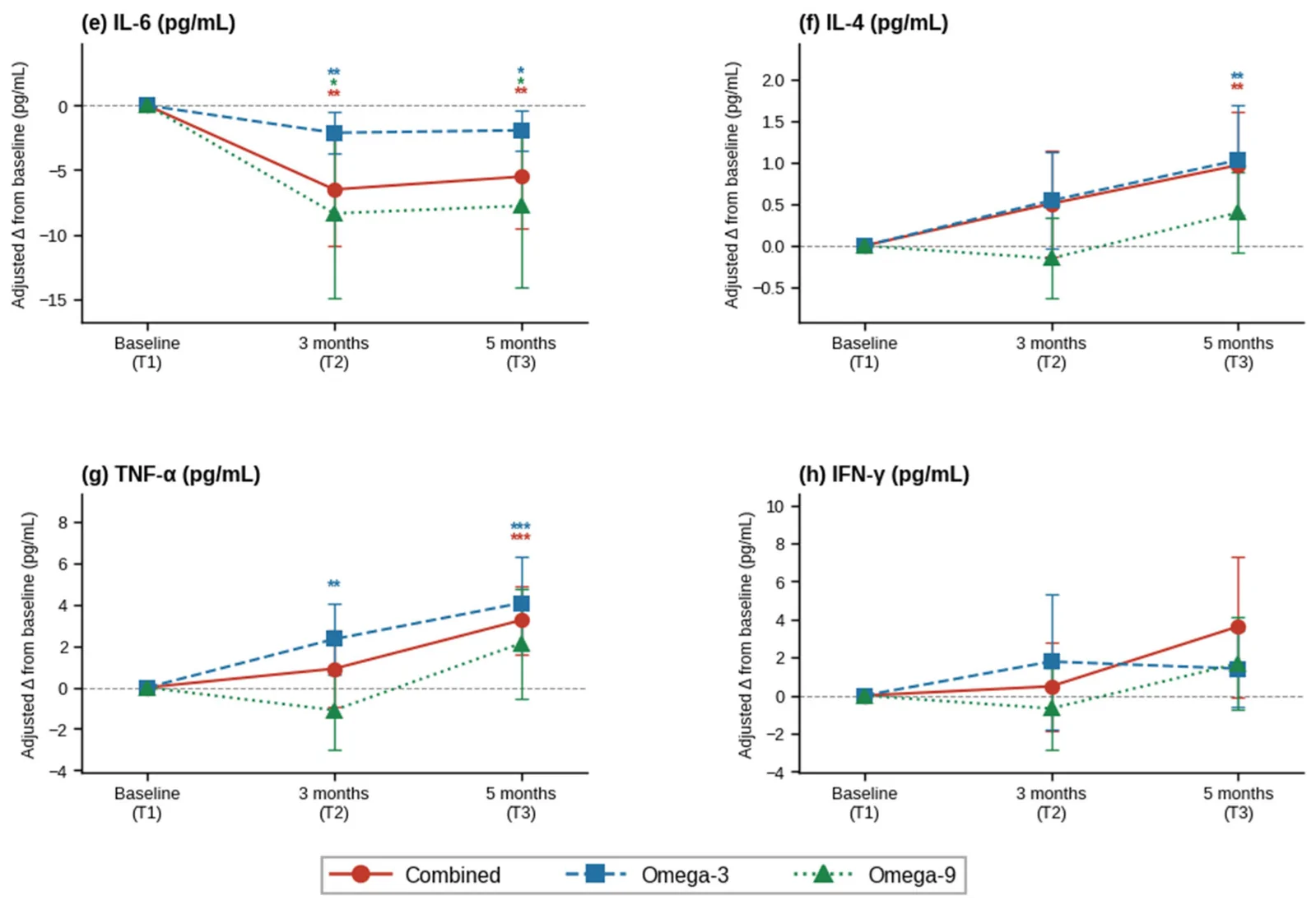
GEE-adjusted mean change from baseline. GEE-adjusted mean change from baseline at each timepoint (unstructured correlation structure; cell-means reparameterization estimating within-group time effects for all three arms simultaneously). Groups are distinguished by three independent visual cues—line color, marker shape, and line style: Combined (solid red circles), Omega-3 (dashed blue squares), and Omega-9 (dotted green triangles). All panels show values in original units, except panel (**d**), which shows leptin on the natural log scale due to upper detection limit censoring (600 ng/mL; see Section 2.4). Adjustment covariates as described in Section 2.5. Significance markers are colored by group and denote the adjusted within-group change from baseline at each timepoint: * *p* < 0.05; ** *p* < 0.01; *** *p* < 0.001. Full within-group contrast estimates (β, 95% CI, and *p*-values) are available in the Supplementary Materials Dataset.

As a sensitivity analysis to assess the robustness of longitudinal findings, generalized estimating equation (GEE) models were fitted for the nine principal outcomes (HOMA-IR, insulin, glucose, IL-6, leptin, IL-4, TNF-α, IFN-γ, and IL-1β), incorporating all three assessment timepoints simultaneously (N = 97). Leptin was modeled on the logarithmic scale. Each model included as fixed predictors the intervention group (reference: Combined), time as a categorical variable (reference: T1), the group × time interaction, age, sex, baseline Z-BMI, the concurrent change in Z-BMI (time-varying covariate), cumulative capsule consumption, and the corresponding baseline outcome value. An exchangeable correlation structure was used as the primary working correlation for GEE models, selected for its parsimony given the balanced three-timepoint design; results were confirmed to be robust to this choice, as a robustness check using an unstructured correlation structure produced virtually identical significance patterns across all nine outcomes (53 of 54 model terms), despite marginally lower quasi-likelihood under the independence model criterion (QIC) values for the unstructured structure (e.g., HOMA-IR: 313.13 vs. 312.29). Full model coefficients are available in the Supplementary Materials Dataset deposited at Zenodo (https://doi.org/10.5281/zenodo.20835542). For graphical representation, a cell-means reparameterization of the GEE model was additionally employed to extract adjusted within-group time contrasts (T1 → T2 and T1 → T3) for each arm simultaneously, as illustrated in Figure 2.

Adiponectin was excluded from statistical analyses because 74% of baseline samples were below the lower limit of detection (0.40 µg/mL). Analyses were performed in R (version 4.6.0; R Foundation for Statistical Computing, Vienna, Austria) and Stata (version 17.0; StataCorp, College Station, TX, USA), with statistical significance set at *p* < 0.05.

## 3. Results

### 3.1. Baseline Characteristics

A total of 97 pediatric participants aged 8–17 years with overweight or obesity and insulin resistance (IR) met eligibility criteria and were included in the analysis: omega-3 (n = 32), combined supplementation (n = 31), and omega-9 (n = 34).

Demographic and clinical characteristics are summarized in Table 1. Overall, the sample was homogeneous, with no statistically significant between-group differences prior to intervention.

Age and sex distribution were comparable across groups (*p* = 0.898 and *p* = 0.849, respectively), with a predominance of females in all three arms (55–62%). Detailed baseline characteristics are presented in Table 1.

No significant baseline differences were identified between groups for any anthropometric variable (weight, height, BMI, Z-BMI, or waist circumference) or for metabolic markers (glucose, insulin, and HOMA-IR).

The baseline inflammatory profile was statistically similar across the three groups (all *p* > 0.05). The sole exception was TNF-α, which differed significantly at baseline (*p* = 0.028), with higher concentrations observed in the omega-9 group [10.6 (7.9–15.6) pg/mL] compared with the omega-3 [7.6 (6.7–10.5) pg/mL] and combined [7.9 (6.4–11.5) pg/mL] groups.

Treatment adherence exceeded 97% across all groups (omega-3: 97.4 ± 4.0%; combined: 97.6 ± 3.3%; omega-9: 98.2 ± 2.5%), with no significant between-group differences (*p* = 0.901). Mild gastrointestinal symptoms (regurgitation or eructation following capsule intake) were reported via the diary card in a subset of participants across the three groups; no participant discontinued treatment due to tolerability concerns, and no other adverse events were recorded.

### 3.2. Intra and Intergroup Longitudinal Changes (Table 2)

#### 3.2.1. Anthropometric Outcomes

Waist circumference percentile decreased significantly across all groups during the intervention and follow-up phases. Significant reductions in Z-BMI were observed in all three groups during the intervention phase. At month 5, persistent reductions in Z-BMI were observed in the combined and omega-9 groups. No significant between-group differences were found in any period.

#### 3.2.2. Metabolic Outcomes

Fasting glucose remained stable throughout follow-up in all groups. During active supplementation, with no significant between-group differences, both the Combined and Omega-9 groups showed significant reductions in fasting insulin and HOMA-IR; the Omega-3 group showed a non-significant reduction in HOMA-IR in the same direction (*p* = 0.074), with no appreciable trend for fasting insulin. After treatment discontinuation, only the Combined group maintained significant reductions in both outcomes (*p* < 0.05), while neither the Omega-3 nor Omega-9 groups showed significant changes at post-intervention follow-up. No significant between-group differences were detected at either timepoint.

#### 3.2.3. Inflammatory Biomarker Response

***Responses shared across groups.*** IL-6 and leptin decreased significantly in all three groups during active supplementation and remained significantly reduced at month 5.

***Intervention-specific responses.*** IL-4 increased progressively across the study period, with significant increases in all groups by month 5 (T3 − T1) and a nominally significant between-group difference at post-intervention follow-up (Kruskal–Wallis, *p* = 0.048). TNF-α increased significantly in the omega-3 group during intervention and remained increased from baseline at month 5 in both the omega-3 and combined groups. IL-1β decreased significantly only in the omega-9 group during the active phase. IFN-γ showed no significant changes at month 3 but modest increases across all groups by month 5. No significant changes were identified for IL-10 or MCP-1/CCL2.

### 3.3. Multivariable Regression Analysis

#### 3.3.1. Metabolic Outcome Predictors

Across all three metabolic models, the corresponding baseline outcome value was the strongest independent predictor of change at both timepoints (month 3: glucose β = −0.61, *p* < 0.001, R^2^ = 0.49; insulin β = −0.49, *p* < 0.001, R^2^ = 0.452; HOMA-IR β = −0.54, *p* < 0.001, R^2^ = 0.525—month 5: glucose β = −0.74, *p* < 0.001, R^2^ = 0.511; insulin β = −0.37, *p* < 0.001, R^2^ = 0.194; HOMA-IR β = −0.42, *p* < 0.001, R^2^ = 0.207), reflecting regression toward less extreme values among children with more severe baseline derangement.

During active intervention, greater concurrent BMI reduction was associated with improvements in all three outcomes (glucose: β = −13.21, *p* = 0.003; insulin: β = −10.6, *p* < 0.001; HOMA-IR: β = −2.68, *p* < 0.001), indicating that adiposity loss was independently associated with metabolic improvement. Higher baseline Z-BMI was additionally associated with attenuated short-term insulin and HOMA-IR reductions (β = 3.38, *p* = 0.001 and β = 0.87, *p* < 0.001, respectively), as was female sex for HOMA-IR reduction (β = 0.96, *p* = 0.007). Capsule consumption showed a non-significant trend toward association with short-term glucose change (β = −0.24, *p* = 0.092). After treatment discontinuation, adiposity and demographic covariates no longer reached significance, and the baseline outcome value remained the only consistent predictor across all three models.

In a sensitivity analysis including recruitment site as a covariate, site was significantly associated with ΔHOMA-IR (β = 0.87, *p* = 0.013), indicating some site-level heterogeneity in metabolic response; however, intervention group coefficients remained essentially unchanged and non-significant (Omega-3: β = 0.59 → 0.61; Omega-9: β = 0.41 → 0.53), confirming that intervention group is not an independent predictor of metabolic response after adjustment for recruitment site. In a further sensitivity analysis including Tanner stage (prepubertal vs. advanced pubertal) as a covariate, Tanner stage was not significantly associated with ΔHOMA-IR (β = 0.51, *p* = 0.153), and intervention group coefficients again remained non-significant in both directions (Omega-3: β = 0.59 → 0.45; Omega-9: β = 0.41 → 0.40), further supporting the robustness of this finding. Notably, intervention group was not independently associated with the metabolic response at either timepoint. Full model coefficients are presented in Table 3.

#### 3.3.2. Inflammatory Outcome Predictors

Across models for both the active-treatment (ΔT2 − T1) and post-intervention follow-up (ΔT3 − T1) periods, the baseline cytokine value was the dominant predictor of inflammatory change (all *p* < 0.001). Baseline Z-BMI was an independent predictor of leptin change at both the active-treatment (ΔT2 − T1: β = 0.444, *p* = 0.015) and post-intervention follow-up (ΔT3 − T1: β = 0.461, *p* = 0.004) timepoints, indicating that children with greater baseline adiposity showed attenuated leptin reductions. Concurrent BMI reduction was independently associated with Δlog IL-4 at both timepoints (ΔT2 − T1: β = 1.05, *p* = 0.006; ΔT3 − T1: β = 0.58, *p* = 0.024), indicating that greater adiposity loss was associated with greater IL-4 increases; it also predicted Δlog TNF-α at post-intervention follow-up (β = −0.526, *p* = 0.004), with greater adiposity loss associated with an attenuated TNF-α increase from baseline. Higher capsule consumption was marginally associated with greater leptin reduction at post-intervention follow-up (β = −0.020, *p* = 0.032), though reverse causality cannot be excluded. Intervention group was not an independent predictor for most inflammatory outcomes, with one exception: omega-9 group membership was associated with a smaller IL-4 increase at post-intervention follow-up relative to the combined group (β = −0.368, *p* = 0.043). This association was not confirmed in the GEE sensitivity model (omega-9 × T3 interaction, *p* = 0.163) and should be interpreted cautiously. Full model coefficients are presented in Table 4.

### 3.4. Sensitivity Analysis: Generalized Estimating Equation (GEE) Models

The following results correspond to the original parameterization (group + time + group × time); adjusted within-group time effects depicted in Figure 2 were derived from a cell-means reparameterization as described in Section 2.5. Generalized estimating equation models incorporating all three assessment timepoints simultaneously confirmed the primary findings. For metabolic outcomes, no significant group × time interactions were identified for glucose, insulin, or HOMA-IR (all *p* > 0.10), indicating that trajectories did not differ significantly between intervention groups. Significant time effects were observed for HOMA-IR and fasting insulin in the combined group at both T2 and T3, whereas glucose remained stable throughout follow-up. For inflammatory outcomes, IL-6 and leptin decreased significantly across all three groups at both timepoints, with the largest absolute reductions observed in the Combined group (all *p* ≤ 0.007); IL-4 increased significantly at month 5 in the Combined and Omega-3 groups (β = 0.97 to 1.05, *p* ≤ 0.002). Across all models, baseline outcome value and concurrent changes in adiposity remained the principal determinants of variation, consistent with the primary analyses. Full model coefficients are presented in the Supplementary Materials Dataset. Of note, the GEE model identified a significant group effect for TNF-α, with the Omega-9 group showing higher overall concentrations relative to the Combined reference across all timepoints (β = 1.254, 95% CI: 0.101–2.407, *p* = 0.033), a finding likely reflecting residual influence of the baseline imbalance in this variable despite adjustment. Adjusted trajectories for all primary and selected secondary outcomes are illustrated in Figure 2.

### 3.5. Sensitivity Analysis: Leptin Detection-Limit Exclusion

To assess the influence of detection-limit censoring on leptin results, all leptin models were re-run excluding participants with baseline values at or above the upper detection limit (n = 18; Combined: 3, Omega-3: 9, Omega-9: 6; remaining n = 79). Combined group reductions remained significant at both timepoints (T1 → T2: β = −0.815, *p* = 0.008; T1 → T3: β = −0.734, *p* < 0.001). Omega-3 reductions were attenuated but persistent (T1 → T3: β = −0.950, *p* = 0.014). The Omega-9 group lost short-term significance at T2 (β = −0.170, *p* = 0.181) but maintained significance at T3 (β = −0.626, *p* < 0.001), consistent with the disproportionate concentration of censored values in this arm.

## 4. Discussion

This prespecified secondary analysis of a triple-blind randomized controlled trial provides new insights into the inflammatory and metabolic effects of omega-3, omega-9, and combined supplementation in pediatric patients with obesity and insulin resistance. Active omega-9 and combined supplementation were associated with short-term reductions in fasting insulin and HOMA-IR, and only the combined treatment maintained a statistically significant within-group reduction in these outcomes at month 5. This should not be interpreted as evidence of superior efficacy relative to the other arms, since direct between-group comparisons were non-significant throughout and treatment group was not an independent predictor of metabolic response in multivariable models; we therefore describe this pattern as within-group persistence of benefit in the Combined arm, rather than differential treatment efficacy. Our longitudinal analysis further revealed an intervention-dependent remodeling of the inflammatory cascade: while markers of chronic lipoinflammation—particularly IL-6 and leptin—decreased consistently across all groups, suggesting a durable anti-inflammatory effect independent of the specific fatty acid administered, cytokines implicated in adipose tissue remodeling (TNF-α and IFN-γ) displayed more complex dynamics [29,30]. The persistent increase from baseline in TNF-α and IFN-γ, observed alongside sustained improvements in insulin resistance and leptin concentrations and without evidence of metabolic deterioration, cannot be mechanistically explained by our data; one plausible but unconfirmed hypothesis is that it reflects adipose tissue immune remodeling rather than a worsening of systemic inflammation, though this interpretation remains speculative (discussed further below) [29].

A central aspect of our findings warrants careful interpretation. Although the Combined group was the only arm to retain a statistically significant within-group reduction in metabolic outcomes at post-intervention follow-up, intervention group was not a predictor of metabolic response for any outcome or period in the multivariable regression models. Instead, baseline metabolic status and concurrent changes in adiposity emerged as the most robust determinants of the observed response. Rather than constituting a contradiction, these results reflect two complementary levels of analysis: at the descriptive level, all three groups showed improvements in inflammatory and metabolic profile under intervention, with within-group persistence at follow-up observed only in the Combined arm; at the level of population-wide determinants, the magnitude of the response was most strongly associated with baseline adiposity and metabolic status rather than treatment group.

When compared with existing evidence, a previous meta-analysis of 9 studies (n = 595) reported a pooled HOMA-IR reduction of −0.38 (95% CI: −0.67 to −0.10) following omega-3 supplementation in pediatric patients with overweight or obesity [12]. The reductions observed in the combined group of our study (median ΔHOMA-IR of −1.28 at T2 − T1 and −1.01 at T3 − T1) substantially exceed this average reported effect. This discrepancy suggests that combined supplementation may be particularly effective in patients with documented IR (HOMA-IR > 3.0), highlighting the importance of baseline phenotypic selection as a critical factor for therapeutic success—an interpretation supported by our regression findings.

To our knowledge, this is among the few pediatric trials evaluating both the active and post-cessation phases of fatty acid supplementation. The marked short-term reduction in fasting insulin and HOMA-IR is consistent with previously described mechanisms by which omega-3 and omega-9 fatty acids modulate proximal insulin signaling, reduce lipotoxicity, and attenuate hepatic steatosis [7,10]. Activation of the GPR120/FFAR4 receptor by EPA and DHA in adipose tissue macrophages and adipocytes has been described to inhibit NF-κB-mediated TLR4 signaling, reduce proinflammatory cytokine production, and enhance GLUT4 translocation [9]. Omega-9 supplementation activates complementary pathways (AMPK, PPARα/γ) that may potentiate the pro-resolving signaling initiated by omega-3 via GPR120; the convergent activation of AMPK by omega-9 and GPR120 signaling by EPA/DHA could generate dual inhibition of the NLRP3 inflammasome, including regulation of IL-1β secretion, which may help explain the within-group persistence of metabolic benefit observed in the Combined arm [9,20,21].

Our multivariable regression analysis adds an important nuance: the efficacy of lipid supplementation appears to be strongly conditioned by the patient’s baseline degree of adiposity, particularly during the active intervention phase. Children with higher baseline Z-BMI scores showed attenuated short-term improvements in insulin sensitivity, suggesting that exogenous functional lipids must act within an already-expanded endogenous fatty acid environment derived from hypertrophic adipose tissue [30]. Likewise, reductions in Z-BMI were independently associated with improvements in glucose, insulin, and HOMA-IR during active intervention, reinforcing the concept that fatty acid supplementation in pediatric obesity should not be interpreted as a standalone intervention, but as a metabolic adjuvant whose efficacy is enhanced when accompanied by active adipose tissue reduction [1,14,30].

In short-term regression models, capsule consumption showed a non-significant trend toward association with glucose change (β = −0.24, *p* = 0.092), which did not reach statistical significance for any metabolic outcome. Accordingly, adherence is reported as a contextual descriptor rather than as evidence of a dose-dependent effect. Reverse causality cannot be excluded, as participants experiencing early metabolic benefit may have maintained higher adherence as a result, not the inverse.

A particularly notable finding was the divergence between the robust decrease in markers of chronic lipoinflammation (IL-6 and leptin) and the fluctuations observed in TNF-α and IFN-γ [4]. One mechanism that may explain the persistence of anti-inflammatory effects after treatment discontinuation involves the biogenesis of specialized pro-resolving mediators (SPMs: resolvins, protectins, maresins) derived from EPA and DHA; their incorporation into membrane phospholipids, with a half-life of weeks, could sustain local SPM production even after supplementation is withdrawn [31].

Although TNF-α and IFN-γ are traditionally classified as proinflammatory mediators, their late increase from baseline in the context of sustained improvements in IR and leptin concentrations, without evidence of metabolic deterioration, is a notable finding. One possible interpretation is that this pattern reflects adipose tissue immune remodeling [2,30,32,33], rather than a worsening of systemic inflammation; however, this interpretation cannot be mechanistically confirmed by our data and remains speculative, as biomarkers were measured systemically and macrophage polarization markers, adipose tissue composition, and specialized pro-resolving mediators were not directly assessed. Confirmation would require studies incorporating direct tissue-level evaluation.

Regarding IL-4, all groups showed progressive and significant increases by month 5, consistent with the absence of a significant overall between-group contrast; this pattern suggests a shared temporal trend independent of the specific fatty acid administered. The nominally significant between-group difference detected at post-intervention follow-up (Kruskal–Wallis, *p* = 0.048) should be interpreted as hypothesis-generating rather than evidence of a differential effect, given the exploratory nature of between-group inflammatory comparisons in this trial. In the context of white adipose tissue remodeling, IL-4 extends beyond its classical role as an anti-inflammatory Th2 cytokine; it activates M2 macrophages in subcutaneous adipose tissue, which secrete catecholamines that promote thermogenesis and beige adipocyte differentiation [34]. This pattern, shared across all three groups, may reflect a common transition toward a reparative microenvironment, independent of the specific fatty acid administered.

### Strengths and Limitations

The strengths of this study include: the randomized, triple-blind design—blinding extended to participants, outcome assessors, and the statistical analysis—minimized selection, performance, and observer bias. The inclusion of both the active intervention and post-cessation follow-up phases allowed acute metabolic responses to be distinguished from the persistence of immunometabolic adaptations, while the simultaneous assessment of metabolic and inflammatory biomarkers provided a comprehensive characterization of treatment response. Finally, GEE models incorporating all three timepoints and intra-individual correlation served as a sensitivity analysis, confirming the principal delta-based findings.

This trial lacked a placebo or untreated arm; all groups received active supplementation plus standardized nutritional counseling, so observed improvements cannot be attributed specifically to the fatty acids rather than to counseling, adiposity reduction, natural course, or regression to the mean, and findings should be interpreted as hypothesis-generating rather than as pharmacological evidence. Z-BMI reduction did not differ between arms (Table 2), but this does not fully resolve the confound given the absence of a placebo. The HOMA-IR > 3.0 eligibility criterion also makes regression to the mean a likely contributor to within-group improvement, consistent with the strong association between baseline value and subsequent change (Table 3 and Table 4). The Guadalajara site was excluded for sample-preservation reasons unrelated to outcome, reducing the analytic sample, though baseline characteristics did not differ from the full randomized cohort (Section 2.1).

Insulin resistance was assessed only via HOMA-IR, a validated indirect surrogate [25]; OGTT or clamp-based measures were not feasible given the trial’s design and pediatric outpatient setting. Body composition relied on anthropometrics alone (BMI, Z-BMI, waist circumference) rather than bioelectrical impedance, DXA, or imaging, so visceral fat—which may more directly mediate the adiposity–insulin resistance relationship—was not directly assessed. Formula B (avocado oil) was not independently verified for fatty acid composition or purity beyond manufacturer specification. Adiponectin was excluded because 74% of baseline samples fell below the assay’s detection limit (0.40 µg/mL). Leptin values at the assay’s upper limit were unevenly distributed across groups (Section 2.4), which may have led to underestimation of the leptin reduction in the Omega-3 group specifically; leptin results should therefore be interpreted conservatively. The baseline TNF-α imbalance was addressed by covariate adjustment.

Pubertal stage differed significantly across arms (χ^2^ = 7.77, *p* = 0.021), likely reflecting chance imbalance given the modest per-arm sample; a sensitivity analysis with Tanner stage as covariate did not alter the non-significant group–response association (Section 3.3.1). Diet and physical activity were not formally quantified beyond standardized counseling, an additional source of unmeasured confounding. Finally, tolerability was assessed via a self/caregiver-reported diary card rather than a validated, severity-graded instrument; it captured the mild gastrointestinal symptoms (regurgitation/eructation) reported here but may under-detect the full spectrum of mild or transient issues, particularly in children.

## 5. Conclusions

All three intervention strategies were associated with within-group improvements in glucose metabolism and inflammatory markers during active supplementation, without significant differences between groups. The Combined group was the only arm to retain a statistically significant within-group reduction in HOMA-IR-defined insulin resistance and fasting insulin after treatment discontinuation; however, adjusted analyses did not demonstrate an independent effect of intervention group, and this finding should not be interpreted as evidence of comparative superiority. These findings support further investigation of omega-3- and omega-9-based supplementation as adjuvant components of comprehensive metabolic management strategies in pediatric obesity.

## Figures and Tables

**Table 1:** Baseline characteristics of obese children with insulin resistance according to treatment group.

Variable	Omega-3 (n = 32)	Combined (n = 31)	Omega-9 (n = 34)	*p*
Anthropometric and demographic				
Age (years)	10.9 (9.5–12.0)	10.2 (9.7–11.6)	10.7 (9.8–11.4)	0.898
Sex, n (% female)	19 (59%)	17 (55%)	21 (62%)	0.849
Weight (kg)	60.6 (51.6–70.7)	55.5 (47.5–65.1)	53.3 (48.0–64.9)	0.198
Height (m)	1.45 (1.41–1.51)	1.41 (1.37–1.49)	1.43 (1.37–1.48)	0.193
BMI (kg/m^2^)	28.0 (25.1–31.5)	27.0 (25.5–31.0)	27.17 (24.23–29.74)	0.260
Z-BMI score	2.9 (2.4–3.4)	2.8 (2.5–3.2)	2.6 (2.4–3.1)	0.288
WC (cm)	87.6 (82.4–94.1)	85.2 (81.5–89.2)	86.7 (80.2–93.0)	0.519
WC (percentile)	94 (87–97)	92 (86–96)	92 (85–97)	0.944
Metabolic markers				
Fasting glucose (mg/dL)	102.7 ± 14.0	97.5 ± 11.1	103.1 ± 14.4	0.183
Fasting insulin (µIU/mL)	22.8 (17.2–29.8)	21.3 (18.3–26.6)	19.4 (16.1–24.4)	0.301
HOMA-IR	5.0 (4.0–6.8)	4.6 (4.0–6.3)	4.3 (3.7–5.7)	0.420
Inflammatory markers and adipokines (pg/mL)				
IL-1β	1.5 (0.9–2.9)	2.8 (0.9–3.2)	1.6 (1.0–3.7)	0.609
IL-4	1.8 (0.6–1.9)	1.8 (0.6–1.9)	1.9 (0.6–3.1)	0.576
IL-6	2.9 (2.0–4.8)	3.9 (2.6–5.9)	4.1 (2.7–10.0)	0.228
IL-10	3.2 (1.2–6.1)	3.2 (2.7–9.8)	3.2 (2.3–4.9)	0.462
IFN-γ	3.2 (0.6–3.2)	3.2 (3.2–4.5)	3.2 (1.9–3.2)	0.137
TNF-α	7.6 (6.7–10.5)	7.9 (6.4–11.5)	10.6 (7.9–15.6)	0.028 *
MCP-1	683.2 (569.7–882.3)	687.3 (583.4–934.4)	717.4 (632.0–912.0)	0.575
Leptin (ng/mL)	30.2 (22.6–600.0)	26.3 (22.8–30.7)	24.1 (20.5–35.4)	0.152
Capsule consumption (%)	97.4 ± 4.0	97.6 ± 3.3	98.2 ± 2.5	0.901

Data presented as mean ± standard deviation (SD) or median (interquartile range, IQR). Sex: n (% female). * *p* < 0.05. Leptin values ≥ 600 ng/mL reflect the upper detection limit; see Section 2.4. Adiponectin excluded; see Section 2.4. Capsule consumption expressed as percentage of target 180 capsules; compared by Kruskal–Wallis test.

**Table 2:** Intra and intergroup longitudinal changes (Δ) by period (N = 97).

Variable	Period	Omega-3 (n = 32)	Combined (n = 31)	Omega-9 (n = 34)	*p* Inter
WC percentile	T2 − T1	−1 (−10; 0) **	−4 (−9; −1) ***	−3 (−10; −1) ***	0.372
	T3 − T1	−2 (−7; 0) **	−4 (−12; −1) ***	−5 (−11; −2) ***	0.209
Z-BMI score	T2 − T1	−0.06 (−0.20; 0.05) *	−0.07 (−0.19; 0.02) *	−0.08 (−0.19; 0.03) *	0.990
	T3 − T1	0.02 (−0.26; 0.14)	−0.08 (−0.36; 0.09) *	−0.04 (−0.29; 0.07) *	0.638
HOMA-IR	T2 − T1	−0.96 (−1.71; 0.58)	−1.28 (−2.34; −0.25) ***	−0.92 (−2.08; 0.24) **	0.348
	T3 − T1	−0.28 (−1.42; 1.03)	−1.01 (−1.69; 0.40) *	−0.32 (−2.28; 0.97)	0.351
Insulin (µIU/mL)	T2 − T1	−3.15 (−8.00; 3.50)	−6.60 (−9.45; −1.55) ***	−4.05 (−8.40; 0.88) *	0.247
	T3 − T1	−1.20 (−5.37; 6.48)	−3.20 (−6.65; 1.10) **	−2.15 (−8.32; 4.25)	0.302
Glucose (mg/dL)	T2 − T1	0.00 (−8.25; 12.25)	3.00 (−4.00; 8.50)	−1.50 (−8.00; 6.00)	0.529
	T3 − T1	1.50 (−11.00; 10.25)	2.00 (−8.00; 7.00)	0.00 (−10.00; 6.75)	0.673
IL-6 (pg/mL)	T2 − T1	−1.29 (−3.06; −0.01) **^,‡^	−2.19 (−3.70; −1.00) ***^,‡^	−2.19 (−6.51; −0.71) ***^,‡^	0.261
	T3 − T1	−0.31 (−3.83; 0.68) *^,‡^	−1.76 (−3.72; −0.15) ***^,‡^	−1.37 (−6.34; −0.18) **^,‡^	0.313
Leptin (ng/mL)	T2 − T1	−7.58 (−544.8; −2.4) ***^,‡^	−10.73 (−15.15; 1.05) **^,‡^	−7.22 (−12.89; 4.28) *^,‡^	0.560
	T3 − T1	−12.51 (−561.07; −5.88) ***^,‡^	−12.66 (−18.58; −8.53) ***^,‡^	−10.09 (−21.96; −2.56) ***^,‡^	0.542
IL-4 (pg/mL)	T2 − T1	0.10 (−0.32; 1.61)	1.27 (−0.32; 1.42) *	0.00 (−1.29; 1.13)	0.155
	T3 − T1	1.24 (−0.01; 1.85) **^,‡^	1.33 (0.60; 1.61) ***^,‡^	0.68 (−0.31; 1.29) *^,‡^	0.048
TNF-α (pg/mL)	T2 − T1	2.43 (−1.49; 3.95) *	0.35 (−2.81; 2.56)	−0.63 (−5.07; 2.41)	0.064
	T3 − T1	3.47 (−0.53; 7.39) **^,‡^	1.92 (0.21; 4.76) **^,‡^	0.94 (−2.80; 5.84)	0.548
IFN-γ (pg/mL)	T2 − T1	0.00 (−1.37; 1.21)	0.00 (−2.31; 1.17)	0.00 (−1.67; 0.78)	0.939
	T3 − T1	1.54 (0.00; 2.76) *^,‡^	0.66 (−1.54; 6.66) *^,‡^	1.31 (0.00; 3.06) **^,‡^	0.997
IL-1β (pg/mL)	T2 − T1	0.00 (−0.64; 1.59)	−0.00 (−2.51; 0.94)	−0.50 (−2.00; 0.04) *	0.127
	T3 − T1	−0.04 (−1.74; 0.92)	−0.41 (−2.56; 0.85)	−0.09 (−0.71; 1.05)	0.684
IL-10 (pg/mL)	T2 − T1	0.90 (−2.33; 2.53)	−0.58 (−2.99; 3.13)	0.00 (−2.01; 2.67)	0.697
	T3 − T1	1.03 (−1.07; 3.66)	−0.29 (−3.67; 2.14)	−0.00 (−2.03; 3.21)	0.284
MCP-1 (pg/mL)	T2 − T1	62.1 (−151.2; 171.0)	19.7 (−178.5; 136.5)	83.1 (−54.1; 191.8)	0.527
	T3 − T1	65.5 (−96.6; 239.6)	67.5 (−28.0; 222.2)	105.6 (−24.8; 328.7)	0.735

Values: median (IQR) of Δ (post—baseline). Intragroup significance by Wilcoxon signed-rank test: * *p* < 0.05; ** *p* < 0.01; *** *p* < 0.001. ^‡^ q < 0.05 (Benjamini–Hochberg FDR correction applied within each variable and period across the three groups; FDR applied to inflammatory biomarkers only). Leptin extreme values in the Omega-3 group reflect upper detection limit concentrations; see Section 2.4.

**Table 3:** Multivariable linear regression models for metabolic response (n = 96).

Panel A. Short-term predictors (ΔT2 − T1)			
**Predictor**	**Δ Glucose**	**Δ Insulin**	**Δ HOMA-IR**
Omega-3 group	1.278 (−3.697, 6.252) 0.611	2.511 (−0.912, 5.934) 0.148	0.598 (−0.168, 1.365) 0.124
Omega-9 group	1.384 (−3.526, 6.293) 0.577	2.437 (−0.956, 5.830) 0.157	0.502 (−0.256, 1.259) 0.192
Age	0.717 (−0.355, 1.789) 0.187	−0.478 (−1.232, 0.276) 0.211	−0.102 (−0.272, 0.067) 0.233
Female	1.573 (−2.785, 5.932) 0.475	**3.132 (0.056, 6.208)** **0.046**	**0.956 (0.273, 1.639)** **0.007**
Baseline Z-BMI	1.733 (−1.202, 4.667) 0.244	**3.385 (1.360, 5.410)** **0.001**	**0.872 (0.424, 1.321)** **<0.001**
Concurrent BMI reduction	**−13.206 (−21.754, −4.659)** **0.003**	**−10.613 (−16.566, −4.660)** **<0.001**	**−2.682 (−4.015, −1.350)** **<0.001**
Baseline outcome value	**−0.607 (−0.764, −0.451)** **<0.001**	**−0.492 (−0.650, −0.334)** **<0.001**	**−0.539 (−0.681, −0.397)** **<0.001**
Capsule consumption	−0.242 (−0.523, 0.040) 0.092	−0.153 (−0.350, 0.043) 0.125	−0.010 (−0.054, 0.034) 0.651
R^2^/n	0.49/96	0.452/96	0.525/96
Panel B. Long-term predictors (ΔT3 − T1)			
Omega-3 group	3.559 (−1.604, 8.722) 0.174	3.530 (−1.090, 8.151) 0.133	0.907 (−0.256, 2.070) 0.125
Omega-9 group	0.059 (−4.982, 5.101) 0.981	1.906 (−2.624, 6.437) 0.405	0.477 (−0.659, 1.614) 0.406
Age	−0.036 (−1.125, 1.053) 0.948	0.173 (−0.824, 1.170) 0.731	0.042 (−0.210, 0.293) 0.743
Female	−1.314 (−5.812, 3.183) 0.563	1.310 (−2.816, 5.437) 0.530	0.457 (−0.573, 1.487) 0.381
Baseline Z-BMI	−0.560 (−3.566, 2.446) 0.712	2.325 (−0.377, 5.028) 0.091	0.656 (−0.016, 1.328) 0.056
Concurrent BMI reduction	1.270 (−5.691, 8.232) 0.718	−5.391 (−11.696, 0.914) 0.093	−1.273 (−2.861, 0.315) 0.115
Baseline outcome value	**−0.744 (−0.906, −0.582)** **<0.001**	**−0.369 (−0.582, −0.156)** **<0.001**	**−0.416 (−0.631, −0.201)** **<0.001**
Capsule consumption	0.017 (−0.165, 0.199) 0.853	−0.079 (−0.244, 0.086) 0.344	−0.010 (−0.051, 0.032) 0.644
R^2^/n	0.511/96	0.194/96	0.207/96

β (95% CI) and *p*-value per cell; bold indicates *p* < 0.05. Panel A: ΔT2 − T1; Panel B: ΔT3 − T1. Adjustment covariates as described in Section 2.5. Concurrent BMI reduction is reverse-coded from ΔZ-BMI (BMI reduction = −ΔZ-BMI), such that positive values reflect greater weight loss; because reductions in these metabolic outcomes reflect clinical improvement, negative coefficients for this variable indicate that greater weight loss is associated with greater improvement. One omega-3 participant excluded for an extreme ΔHOMA-IR value (n = 96). HOMA-IR: homeostatic model assessment of insulin resistance.

**Table 4:** Multivariable linear regression models for inflammatory outcomes (log-transformed; n = 96).

Panel A. Short-term predictors (ΔT2 − T1)				
**Predictor**	**Δlog IL-6**	**Δlog TNF-α**	**Δlog Leptin**	**Δlog IL-4**
Omega-3 group	0.048 (−0.394, 0.491) 0.828	0.136 (−0.095, 0.368) 0.245	0.356 (−0.218, 0.930) 0.221	−0.083 (−0.502, 0.336) 0.695
Omega-9 group	0.063 (−0.370, 0.496) 0.772	−0.007 (−0.240, 0.226) 0.953	0.407 (−0.153, 0.968) 0.152	−0.385 (−0.798, 0.029) 0.068
Age	0.051 (−0.045, 0.146) 0.295	−0.018 (−0.068, 0.033) 0.495	0.055 (−0.071, 0.181) 0.386	−0.070 (−0.162, 0.022) 0.135
Female sex	0.147 (−0.240, 0.534) 0.453	−0.152 (−0.356, 0.053) 0.144	0.236 (−0.282, 0.755) 0.368	−0.273 (−0.644, 0.098) 0.147
Baseline Z-BMI	−0.024 (−0.281, 0.233) 0.853	−0.073 (−0.211, 0.065) 0.298	**0.444 (0.088, 0.800)** **0.015**	−0.108 (−0.352, 0.137) 0.384
Concurrent BMI reduction	−0.205 (−0.967, 0.557) 0.595	0.178 (−0.223, 0.579) 0.380	−0.837 (−1.822, 0.148) 0.095	**1.050 (0.316, 1.785)** **0.006**
Capsule consumption	0.003 (−0.023, 0.028) 0.845	0.004 (−0.009, 0.017) 0.567	0.011 (−0.021, 0.044) 0.499	−0.017 (−0.041, 0.007) 0.158
Baseline cytokine value	**−0.976 (−1.128, −0.824) <0.001**	**−0.699 (−0.885, −0.514)** **<0.001**	**−0.807 (−1.008, −0.605)** **0.001**	**−0.821 (−1.027, −0.616)** **<0.001**
R^2^/n	0.679/96	0.475/96	0.45/96	0.483/96
Panel B. Long-term predictors (ΔT3 − T1)				
**Predictor**	**Δlog IL-6**	**Δlog TNF-α**	**Δlog Leptin**	**Δlog IL-4**
Omega-3 group	−0.202 (−0.599, 0.194) 0.313	0.028 (−0.235, 0.291) 0.832	−0.266 (−0.775, 0.243) 0.301	−0.268 (−0.632, 0.097) 0.148
Omega-9 group	0.046 (−0.339, 0.431) 0.813	0.108 (−0.154, 0.370) 0.413	−0.117 (−0.608, 0.374) 0.637	**−0.368 (−0.723, −0.012)** **0.043**
Age	0.044 (−0.040, 0.128) 0.304	0.032 (−0.024, 0.089) 0.259	0.003 (−0.106, 0.112) 0.959	−0.042 (−0.120, 0.036) 0.292
Female sex	0.085 (−0.261, 0.432) 0.626	−0.092 (−0.322, 0.139) 0.431	0.272 (−0.184, 0.729) 0.239	0.216 (−0.104, 0.537) 0.183
Baseline Z-BMI	0.061 (−0.166, 0.289) 0.593	0.086 (−0.069, 0.241) 0.275	**0.461 (0.150, 0.772)** **0.004**	0.130 (−0.079, 0.340) 0.220
Concurrent BMI reduction	0.167 (−0.371, 0.705) 0.539	**−0.526 (−0.883, −0.168)** **0.004**	−0.103 (−0.787, 0.580) 0.765	**0.582 (0.078, 1.085)** **0.024**
Capsule consumption	−0.003 (−0.017, 0.012) 0.723	0.007 (−0.003, 0.016) 0.163	**−0.020 (−0.038, −0.002)** **0.032**	−0.001 (−0.014, 0.012) 0.931
Baseline cytokine value	**−0.857 (−0.990, −0.723)** **<0.001**	**−0.796 (−1.006, −0.586)** **<0.001**	**−1.001 (−1.180, −0.822)** **<0.001**	**−0.791 (−0.970, −0.612)** **<0.001**
R^2^/n	0.661/96	0.477/96	0.647/96	0.505/96

β (95% CI) and *p*-value per cell; bold indicates *p* < 0.05. Outcomes are Δlog(cytokine), i.e., proportional change. Panel A: ΔT2 − T1; Panel B: ΔT3 − T1. Adjustment covariates as in Table 3, plus baseline log-transformed cytokine value. Concurrent BMI reduction is reverse-coded from ΔZ-BMI, as in Table 3; interpretation of direction (improvement vs. increase) depends on the biomarker. Leptin log-transformed; see Section 2.4. Same exclusion as Table 3 (n = 96).

## Data Availability

The analytical results (GEE model coefficients and within-group contrast estimates) supporting the findings of this study are publicly available at Zenodo (https://doi.org/10.5281/zenodo.20835542). The individual-level dataset cannot be made publicly available due to ethical and privacy restrictions associated with pediatric patient data but is available on request from the corresponding author.

## Supplementary Materials

The following supporting information can be downloaded at: https://www.mdpi.com/article/10.3390/nu18162644/s1, Table S1: GEE Sensitivity Analysis—Original Parameterization; Table S2: Within-Group Adjusted Time Contrasts—Cell-Means Reparametrization.

## Abbreviations

The following abbreviations are used in this manuscript:

RCT	Randomized controlled trial
IR	Insulin resistance
HOMA-IR	Homeostatic model assessment of insulin resistance
BMI	Body mass index
Z-BMI	Body mass index z-score
WC	Waist circumference
PUFA	Polyunsaturated fatty acid
MUFA	Monounsaturated fatty acid
EPA	Eicosapentaenoic acid
DHA	Docosahexaenoic acid
IL-1β	Interleukin-1 beta
IL-4	Interleukin-4
IL-6	Interleukin-6
IL-10	Interleukin-10
IFN-γ	Interferon gamma
TNF-α	Tumor necrosis factor alpha
MCP-1	Monocyte chemoattractant protein-1
GEE	Generalized estimating equations
QIC	Quasi-likelihood under the independence model criterion
FDR	False discovery rate
LOD	Limit of detection
VIF	Variance inflation factor
MASLD	Metabolic dysfunction-associated steatotic liver disease
HIMFG	Hospital Infantil de México Federico Gómez
WHO	World Health Organization
SD	Standard deviation
IQR	Interquartile range
SPM	Specialized pro-resolving mediator
CI	Confidence interval
