# Supplementary material for: Effects of Omega-3 and Omega-9 Fatty Acid Supplementation on HOMA-IR and Inflammatory Markers in Children with Obesity and Insulin Resistance: A Triple-Blind RCT with Post-Intervention Follow-Up
Source: Nutrients. 2026 Aug 13;18(16):2644. doi: 10.3390/nu18162644 (PMC13516122; doi:10.3390/nu18162644)
Supplement: Supplementary file 1 [file nutrients-18-02644-s001.zip › nutrients-4430194-supplementary.pdf]

Supplementary Dataset

Table S1. GEE Sensitivity Analysis — Original Parameterization

Model specification: outcome ~ group + time + group×time + covariates; Combined group and T1 as references. Exchangeable correlation structure. N = 97 for all models. Bold indicates $p < 0.05$.

Panel A. Metabolic outcomes

Predictor	HOMA-IR	Insulin (μIU/mL)	Glucose (mg/dL)
Group effects			
Omega-3 group	+0.021 (−0.407, 0.449) p = 0.924	−0.118 (−1.652, 1.415) p = 0.880	+2.410 (−0.569, 5.388) p = 0.113
Omega-9 group	−0.040 (−0.395, 0.316) p = 0.827	−0.345 (−1.735, 1.045) p = 0.627	+2.642 (−0.304, 5.588) p = 0.079
Time effects (Combined as reference)			
T1→T2 (Combined)	**−1.416 (−2.085, −0.747) p < 0.001**	**−6.010 (−8.929, −3.092) p < 0.001**	+3.050 (−0.938, 7.039) p = 0.134
T1→T3 (Combined)	**−0.751 (−1.483, −0.019) p = 0.044**	**−2.790 (−5.352, −0.228) p = 0.033**	+1.086 (−2.367, 4.540) p = 0.538
Group × Time interactions			
Omega-3 × T2	+0.823 (−0.307, 1.953) p = 0.153	+3.549 (−0.798, 7.897) p = 0.110	−1.667 (−7.621, 4.287) p = 0.583
Omega-3 × T3	+0.599 (−0.708, 1.906) p = 0.369	+2.622 (−2.346, 7.589) p = 0.301	−1.164 (−7.241, 4.913) p = 0.707
Omega-9 × T2	+0.627 (−0.221, 1.475) p = 0.147	+3.104 (−0.632, 6.841) p = 0.103	−3.037 (−9.289, 3.216) p = 0.341
Omega-9 × T3	+0.465 (−0.565, 1.494) p = 0.376	+2.108 (−1.787, 6.003) p = 0.289	−4.258 (−10.824, 2.308) p = 0.204
Adjustment covariates			
Age (years)	−0.017 (−0.109, 0.074) p = 0.714	−0.105 (−0.475, 0.264) p = 0.577	+0.101 (−0.497, 0.699) p = 0.740
Female sex	**+0.483 (0.013, 0.954) p = 0.044**	+1.497 (−0.304, 3.299) p = 0.103	+0.031 (−2.376, 2.437) p = 0.980
Baseline Z-BMI	**+0.516 (0.180, 0.852) p = 0.003**	**+1.871 (0.518, 3.223) p = 0.007**	+0.082 (−1.432, 1.596) p = 0.916
Concurrent ΔZ-BMI	**+1.623 (0.508, 2.739) p = 0.004**	**+6.888 (2.678, 11.097) p = 0.001**	+3.101 (−2.076, 8.277) p = 0.240
Capsule consumption (%)	−0.027 (−0.078, 0.023) p = 0.285	−0.134 (−0.301, 0.033) p = 0.115	−0.024 (−0.126, 0.077) p = 0.638
Baseline outcome value	**+0.687 (0.575, 0.799) p < 0.001**	**+0.711 (0.619, 0.804) p < 0.001**	**+0.536 (0.449, 0.623) p < 0.001**
**Exchangeable ρ / n**	**−0.039 / 97**	**−0.050 / 97**	**0.001 / 97**

Each cell: β (95% CI) p-value. "Time: T1→T2 (Combined)" and "Time: T1→T3 (Combined)": adjusted change from baseline in the Combined group. Group×Time coefficients: additional change in Omega-3 or Omega-9 relative to Combined. "Exch. ρ": estimated exchangeable correlation parameter. No group×time interaction reached significance for metabolic outcomes.

Panel B. Inflammatory outcomes

Predictor	IL-6 (pg/mL)	Leptin (log ng/mL)	IL-4 (pg/mL)	TNF-α (pg/mL)	IFN-γ (pg/mL)	IL-1β (pg/mL)
Group effects						
Omega-3 group	−2.724 (−5.753, 0.305) p = 0.078	+0.335 (−0.018, 0.689) p = 0.063	−0.069 (−0.411, 0.272) p = 0.690	−0.280 (−1.068, 0.507) p = 0.486	−0.099 (−0.665, 0.467) p = 0.732	−0.339 (−1.697, 1.019) p = 0.625
Omega-9 group	+1.490 (−3.640, 6.620) p = 0.569	+0.138 (−0.180, 0.457) p = 0.395	+0.038 (−0.318, 0.395) p = 0.833	**+1.254 (0.101, 2.407) p = 0.033**	+0.213 (−0.394, 0.820) p = 0.492	−0.133 (−1.288, 1.022) p = 0.821
Time effects (Combined as reference)						
T1→T2 (Combined)	**−6.512 (−10.895, −2.130) p = 0.004**	**−0.878 (−1.445, −0.311) p = 0.002**	+0.505 (−0.128, 1.137) p = 0.118	+0.904 (−0.949, 2.757) p = 0.339	+0.500 (−1.812, 2.812) p = 0.671	−0.867 (−2.298, 0.563) p = 0.235
T1→T3 (Combined)	**−5.534 (−9.541, −1.527) p = 0.007**	**−0.856 (−1.196, −0.515) p < 0.001**	**+0.968 (0.330, 1.606) p = 0.003**	**+3.238 (1.567, 4.908) p < 0.001**	+3.689 (−0.040, 7.418) p = 0.053	−1.138 (−2.374, 0.098) p = 0.071
Group × Time interactions						
Omega-3 × T2	+4.398 (−0.205, 9.000) p = 0.061	−0.144 (−0.854, 0.565) p = 0.690	+0.038 (−0.800, 0.877) p = 0.929	+1.437 (−1.043, 3.918) p = 0.256	+1.284 (−2.862, 5.430) p = 0.544	+0.272 (−2.257, 2.800) p = 0.833
Omega-3 × T3	+3.599 (−0.651, 7.849) p = 0.097	**−0.813 (−1.562, −0.065) p = 0.033**	+0.059 (−0.840, 0.958) p = 0.898	+0.837 (−1.895, 3.570) p = 0.548	−2.243 (−6.209, 1.722) p = 0.267	−0.054 (−2.441, 2.332) p = 0.964
Omega-9 × T2	−1.858 (−9.732, 6.016) p = 0.644	+0.242 (−0.491, 0.975) p = 0.518	−0.657 (−1.442, 0.129) p = 0.101	−2.013 (−4.640, 0.614) p = 0.133	−1.148 (−4.256, 1.959) p = 0.469	−0.179 (−1.968, 1.609) p = 0.844
Omega-9 × T3	−2.261 (−9.710, 5.187) p = 0.552	−0.311 (−0.892, 0.269) p = 0.293	−0.569 (−1.368, 0.230) p = 0.163	−1.113 (−4.229, 2.003) p = 0.484	−1.949 (−6.070, 2.173) p = 0.354	+0.637 (−0.990, 2.264) p = 0.443
Adjustment covariates						
Age (years)	+0.089 (−0.009, 0.188) p = 0.074	+0.021 (−0.037, 0.079) p = 0.478	−0.002 (−0.104, 0.101) p = 0.976	+0.082 (−0.195, 0.360) p = 0.560	−0.288 (−0.888, 0.311) p = 0.345	−0.015 (−0.103, 0.074) p = 0.747
Female sex	+0.259 (−0.192, 0.710) p = 0.261	+0.163 (−0.000, 0.326) p = 0.050	+0.094 (−0.216, 0.404) p = 0.552	−0.431 (−1.855, 0.992) p = 0.553	−0.858 (−2.401, 0.684) p = 0.275	+0.324 (−0.163, 0.811) p = 0.193
Baseline Z-BMI	+0.050 (−0.274, 0.373) p = 0.763	**+0.297 (0.142, 0.451) p < 0.001**	+0.052 (−0.134, 0.239) p = 0.581	+0.245 (−0.652, 1.143) p = 0.592	+0.259 (−1.065, 1.582) p = 0.702	−0.051 (−0.296, 0.195) p = 0.685
Concurrent ΔZ-BMI	+0.250 (−1.490, 1.991) p = 0.778	+0.411 (−0.052, 0.873) p = 0.082	**−1.004 (−1.699, −0.308) p = 0.005**	**+3.531 (0.284, 6.778) p = 0.033**	+2.466 (−1.546, 6.478) p = 0.228	+0.193 (−0.903, 1.289) p = 0.730
Capsule consumption (%)	−0.007 (−0.056, 0.042) p = 0.777	**−0.013 (−0.022, −0.004) p = 0.004**	+0.003 (−0.012, 0.019) p = 0.675	**+0.064 (0.010, 0.119) p = 0.020**	+0.016 (−0.058, 0.091) p = 0.666	+0.015 (−0.006, 0.037) p = 0.156
Baseline outcome value	**+0.352 (0.322, 0.381) p < 0.001**	**+0.394 (0.299, 0.488) p < 0.001**	**+0.503 (0.402, 0.605) p < 0.001**	**+0.594 (0.391, 0.796) p < 0.001**	**+0.924 (0.718, 1.129) p < 0.001**	**+0.461 (0.347, 0.575) p < 0.001**
**Exchangeable ρ / n**	**−0.447 / 97**	**−0.083 / 97**	**0.094 / 97**	**0.200 / 97**	**0.154 / 97**	**−0.192 / 97**

Each cell: β (95% CI) p-value. Leptin log-transformed; remaining inflammatory biomarkers in original units. Omega-3×T3 interaction for leptin (p = 0.033) interpreted with caution given upper detection-limit censoring (600 ng/mL). TNF-α group effect for Omega-9 (p = 0.033) likely reflects residual influence of baseline imbalance despite covariate adjustment. See Section 2.5 of the main manuscript for full covariate definitions.

Table S2. Within-Group Adjusted Time Contrasts — Cell-Means Reparametrization

Source of significance markers in Figure 2 of the main manuscript. Model specification: outcome ~ grp_t + covariates, where grp_t is a single nine-level factor encoding all group×timepoint combinations (Combined_T1 – Omega9_T3). Adjusted within-group changes from baseline were extracted as post-hoc linear contrasts of the estimated marginal means using the emmeans package (R 4.6.0). Covariates identical to Table 1. N = 97; exchangeable correlation structure. Red bold = $p < 0.05$.

Outcome	Period	Combined		Omega-3		Omega-9	
		β (95% CI)	p	β (95% CI)	p	β (95% CI)	p
**Insulin (μIU/mL)**	T1→T2	**-6.014 (-8.932, -3.096)**	**p < 0.001**	-2.468 (-5.817, 0.882)	p = 0.149	**-2.910 (-5.338, -0.483)**	**p = 0.019**
	T1→T3	**-2.800 (-5.372, -0.227)**	**p = 0.033**	-0.180 (-4.777, 4.417)	p = 0.939	-0.699 (-3.812, 2.413)	p = 0.660
**Glucose (mg/dL)**	T1→T2	+3.059 (-0.934, 7.051)	p = 0.133	+1.376 (-3.091, 5.843)	p = 0.546	+0.032 (-4.837, 4.900)	p = 0.990
	T1→T3	+1.119 (-2.336, 4.574)	p = 0.526	-0.065 (-5.096, 4.966)	p = 0.980	-3.149 (-8.844, 2.546)	p = 0.279
**HOMA-IR**	T1→T2	**-1.417 (-2.086, -0.747)**	**p < 0.001**	-0.595 (-1.517, 0.327)	p = 0.206	**-0.789 (-1.354, -0.224)**	**p = 0.006**
	T1→T3	**-0.751 (-1.484, -0.018)**	**p = 0.044**	-0.154 (-1.330, 1.023)	p = 0.798	-0.288 (-1.103, 0.526)	p = 0.488
**Leptin (log ng/mL)**	T1→T2	**-0.876 (-1.444, -0.309)**	**p = 0.003**	**-1.023 (-1.453, -0.593)**	**p < 0.001**	**-0.633 (-1.092, -0.174)**	**p = 0.007**
	T1→T3	**-0.851 (-1.194, -0.508)**	**p < 0.001**	**-1.667 (-2.357, -0.977)**	**p < 0.001**	**-1.163 (-1.641, -0.685)**	**p < 0.001**
**IL-6 (pg/mL)**	T1→T2	**-6.507 (-10.890, -2.124)**	**p = 0.004**	**-2.116 (-3.699, -0.532)**	**p = 0.009**	**-8.360 (-14.963, -1.757)**	**p = 0.013**
	T1→T3	**-5.514 (-9.526, -1.503)**	**p = 0.007**	**-1.925 (-3.471, -0.378)**	**p = 0.015**	**-7.778 (-14.125, -1.431)**	**p = 0.016**
**IL-4 (pg/mL)**	T1→T2	+0.505 (-0.127, 1.137)	p = 0.117	+0.543 (-0.035, 1.120)	p = 0.065	-0.152 (-0.634, 0.331)	p = 0.537
	T1→T3	**+0.968 (0.331, 1.605)**	**p = 0.003**	**+1.027 (0.369, 1.684)**	**p = 0.002**	+0.399 (-0.086, 0.885)	p = 0.107
**TNF-α (pg/mL)**	T1→T2	+0.908 (-0.942, 2.759)	p = 0.336	**+2.340 (0.595, 4.084)**	**p = 0.009**	-1.100 (-2.987, 0.787)	p = 0.253
	T1→T3	**+3.255 (1.587, 4.922)**	**p < 0.001**	**+4.084 (1.812, 6.355)**	**p < 0.001**	+2.140 (-0.515, 4.795)	p = 0.114
**IFN-γ (pg/mL)**	T1→T2	+0.484 (-1.828, 2.797)	p = 0.682	+1.789 (-1.756, 5.334)	p = 0.323	-0.680 (-2.823, 1.464)	p = 0.534
	T1→T3	+3.629 (-0.077, 7.336)	p = 0.055	+1.414 (-0.618, 3.447)	p = 0.173	+1.687 (-0.755, 4.130)	p = 0.176

β: unstandardized regression coefficient representing the adjusted mean change from baseline (T1) at the specified timepoint. 95% CI: asymptotic Wald confidence interval. Leptin modeled on the natural log scale; all other outcomes in original units. p-values from z-tests on GEE sandwich standard errors. Statistical significance threshold: $p < 0.05$ (two-sided). Cross-validation against unadjusted Wilcoxon signed-rank tests confirmed concordance for 43/48 contrasts (90%); discordant cases (all involving IL-4 or IFN-γ at T3) are attributable to covariate adjustment attenuating effects explained by baseline adiposity.

Individual-level patient data are not included in this dataset due to ethical and privacy restrictions associated with pediatric patient data. Data are available on request from the corresponding author.
